# Obesogens: a unifying theory for the global rise in obesity

**DOI:** 10.1038/s41366-024-01460-3

**Published:** 2024-01-11

**Authors:** Jerrold J. Heindel, Robert H. Lustig, Sarah Howard, Barbara E. Corkey

**Affiliations:** 1 Healthy Environment and Endocrine Disruptor Strategies (HEEDS), Bozeman, MT 59715 USA; 2 Department of Pediatrics and Institute for Health Policy Studies, University of California, San Francisco, CA 94143 USA; 3 Department of Medicine, Boston University, Chobanian and Avedisian School of Medicine, Boston, MA 02118 USA

**Keywords:** Obesity, Hormones

## Abstract

Despite varied treatment, mitigation, and prevention efforts, the global prevalence and severity of obesity continue to worsen. Here we propose a combined model of obesity, a unifying paradigm that links four general models: the energy balance model (EBM), based on calories as the driver of weight gain; the carbohydrate-insulin model (CIM), based on insulin as a driver of energy storage; the oxidation-reduction model (REDOX), based on reactive oxygen species (ROS) as a driver of altered metabolic signaling; and the obesogens model (OBS), which proposes that environmental chemicals interfere with hormonal signaling leading to adiposity. We propose a combined OBS/REDOX model in which environmental chemicals (in air, food, food packaging, and household products) generate false autocrine and endocrine metabolic signals, including ROS, that subvert standard regulatory energy mechanisms, increase basal and stimulated insulin secretion, disrupt energy efficiency, and influence appetite and energy expenditure leading to weight gain. This combined model incorporates the data supporting the EBM and CIM models, thus creating one integrated model that covers significant aspects of all the mechanisms potentially contributing to the obesity pandemic. Importantly, the OBS/REDOX model provides a rationale and approach for future preventative efforts based on environmental chemical exposure reduction.

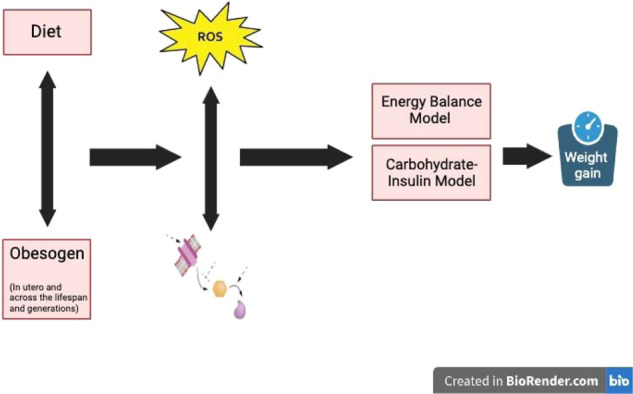

## Introduction and Background

Obesity continues to increase at an alarming rate across the globe despite an increase in the number of diets and drugs [1]. The etiology of obesity is still not understood, as evidenced by the following statements from recent articles:

1. In 2017, an Endocrine Society Scientific Statement [2] noted, “The current lack of consensus regarding obesity pathogenesis has resulted in competing and poorly justified claims both from within and outside the scientific community. These inconsistencies erode public trust and confidence in the scientific process concerning obesity and its treatment, further supporting nonscientific ideologies and products.”
2. A recent perspective noted that we do not have a clear explanation for the obesity epidemic [3]. Notably, the national data do not support higher energy consumption as a driver of the obesity epidemic since 2000. “This lack of adequate attention and investment in understanding the root causes of the obesity epidemic … may at least partly owe to the belief that the foundational causes are already known” [3].
3. A recent scientific meeting organized at the Royal Society in London by Profs. Speakman, Sørensen, Hall, and Allison focused on “Causes of obesity: theories, conjectures and evidence” [4]. Despite numerous symposia, guidelines, and punditry, the attendees were no closer to a unifying theory for the global rise in obesity.

## Ontogeny of obesity

Obesity is a neuroendocrine disease [2]. Body weight is highly regulated by various systems and hormones from many tissues integrated by the brain to regulate food intake and metabolism [5]. Key questions include, what has changed over the last 50 years that led to the obesity epidemic? What has been imposed on or removed from society that led to the obesity epidemic?

Before examining the various models of obesity, it is essential to understand when obesity starts (ontogeny), as that aspect of etiology must be integrated into any model. Obesity, like other non-communicable diseases, can have at least some of its origins *in utero* and early childhood and may manifest itself at any time across the lifespan. Both under and over-nutrition *in utero* are associated with obesity in the offspring later in life [6–10]. Mothers or fathers who are overweight during pregnancy may have overweight offspring [11]. In a rodent study, maternal exercise during pregnancy promoted physical activity in adult offspring, suggesting that the propensity to exercise may also be programmed during development [12]. The strongest perinatal predictor of childhood obesity is reported to be maternal pre-pregnancy obesity [13]. In a rodent study, gestational exposure to a Western diet predisposes to a high fat and sugar diet in later life to promulgate obesity [14]. Developmental programming can also affect intergenerational obesity in humans [15, 16], and transgenerational epigenetic inheritance of obesity is seen in animal models [17]. Altered epigenetic regulation of gene expression during development due to nutrition, stress, or environmental chemicals can interfere with the control of food intake and metabolism, including metabolic efficiency via effects on the development of the adipose tissue, pancreas, liver, gastrointestinal tract, brain and/or muscle, thereby resulting in an altered body weight set point or sensitivity for developing obesity across the lifespan and generations [18]. *In utero* and early development may be a highly sensitive time for the programming of fat storage due to permanent effects on gene expression and adipose tissue differentiation. Consequently, nutrition, stress, and environmental chemicals can all have the potential to alter metabolic signaling at this stage, leading to excessive adipose tissue growth and energy storage throughout life.

## Current models of obesity

Two of the major models of obesity include the energy balance model (EBM) reviewed in [5, 19], which emphasizes overeating and sedentary activity, and the carbohydrate-insulin model (CIM) reviewed in [20], which emphasizes energy storage due to hyperinsulinemia’s effect on adipocytes. The reduction-oxidation model (REDOX) is an additional lesser-known model reviewed in [21, 22]. The REDOX model emphasizes that many substances, including processed foods and environmental exposures, can cause obesity by generating false and misleading information about energy status. This misinformation is driven by changes in the oxidation-reduction potential of metabolites that circulate and communicate to organs throughout the body. A fourth model, the obesogen model (OBS) reviewed in [18], posits that exposure to environmental chemicals, especially during critical developmental periods, but also across the lifespan, can affect long-term metabolism via hormonal changes, increasing susceptibility to obesity.

Here we discuss these four models in more detail. Each model focuses on a specific aspect of obesity: neural control and calories (EBM); carbohydrates and insulin (CIM); metabolic oxidation-reduction mismatches (REDOX); and developmental exposures to environmental stimuli (OBS). Each model is usually presented as an exclusive and non-overlapping archetype responsible for the increase in obesity; however, below, we propose a more integrated approach. We describe, in turn, each model, the integration of the OBS and REDOX models, and finally propose that this OBS/REDOX model can account for much of the data that support both the EBM and CIM models.

## The Energy Balance Model (EBM)

According to the EBM, obesity is a disorder of energy balance. Overweight and obesity result from a chronic imbalance between energy intake and expenditure (21, 25, 26); we gain weight because we eat more, burn fewer calories, or both. The EBM proposes that the brain is the primary organ responsible for body weight regulation via the integration of internal and external signals by mediators not yet defined and that disruption of normal signals leads to overeating and obesity. In this model, it is food intake that needs to be controlled. The EBM notes that consuming ultra-processed food (UPF) causes overeating, increasing adiposity, insulin resistance, consequent insulin compensatory secretion, and resultant weight gain [23]. Recent additions to the EBM include other gastrointestinal hormones (e.g., glucagon-like peptide 1 (GLP-1), peptide YY_3-36_ (PYY), and gastric inhibitory polypeptide (GIP)), all of which reduce acute food intake [24]. GLP-1 acts centrally [25] and peripherally [26] to inhibit food intake. Indeed, newer GLP-1 analogs have become primary therapies for T2D and obesity [27]. However, it should also be noted that GLP-1 analogs may also have untoward side-effects by delaying gastric emptying, leading to nausea, vomiting, and gastroparesis [28]. These side-effects may be part of the mechanism for the weight reduction, as demonstrated by the loss of equal amounts of muscle and fat, consistent with anorexia and/or starvation [29].

The gut microbiome may also support the EBM to predispose to obesity. Animal studies argue that changes in the microbiome increase energy availability by increasing energy harvest efficiency [30]. Several investigators have demonstrated changes in the human microbiome, paralleling changes in the diet [31–33] suggesting that one mechanism of diet-induced obesity may be through microbiome-promotion of altered energy harvesting [34]. Animal models have also provided evidence that a “predisposed” microbiome might increase both energy intake (via central mechanisms) and energy absorption (via gastrointestinal mechanisms) to contribute to obesity [35]. However, while changes in diet (and therefore by inference obesogens) have effects on the gut microbiota, there are currently no compelling data thus far that differentiates between consequence and cause. Furthermore, human data addressing this mechanism have been somewhat inconsistent [36]. “Randomized controlled trials of microbiota transfer in human participants have not shown effects on body weight. With a more critical reading, early studies did not show as large an effect as first appeared and later research, including human trials, has failed to support a role of the gut microbiota in shaping body weight” [37].

The EBM proposes that achieving a stable weight is as simple as balancing energy intake versus expenditure; however, a recent review [38] concluded that weight stability is much more complex. Most individuals’ experimentally induced weight gain or loss has no lasting effects. The original weight is rapidly re-established when the controlled feeding experiment ends [39]. In addition, these studies documented that many more calories than predicted were needed to gain weight. Conversely, a much more significant caloric decrease was required than expected to lose weight, indicating a strong biochemical regulatory mechanism for weight maintenance [40].

Exercise has never been shown to strongly modulate body weight, possibly due to compensatory regulation of energy efficiency and the repartitioning of fat into muscle, likely due to growth hormone secretion [41]. It should be noted that the amount of energy expenditure caused by increased physical activity does not translate directly to weight loss, since if it did, people would lose more weight than they do in trials in which physical activity is increased under close supervision [42].

The EBM does not explain why numerous animal species (both in the wild, near human populations, and in captivity with controlled diets) have all gained weight over the past 25 years [43]. In addition, since 2000, obesity rates have increased while energy intake decreased and energy expenditure increased [3]. The EBM also does not explain why, for a given caloric intake or physical activity, BMI was higher in 2006 than in 1988 [44]. Lastly, the EBM does not address how diet or environmental exposures during development influence later-life obesity.

## The Carbohydrate-Insulin Model (CIM)

The obesity epidemic in the U.S. temporally coincided with the food industry and the federal government’s promotion in the 1970s of low-fat diets and the resulting increased intake of refined carbohydrates and fructose-containing sweeteners, reviewed in [45]. This change was based on the epidemiologic correlation of dietary fat, low-density lipoprotein, cholesterol, and cardiovascular mortality [46]. However, the inevitable result of this paradigm change was increased carbohydrate consumption with induced insulin response, increased energy deposition into adipose tissue, with increased obesity and related chronic non-communicable diseases.

The CIM posits that a diet high in rapidly digestible carbohydrates causes an elevated insulin response that stimulates lipoprotein lipase (LPL) and suppresses the adrenergic system and lipolysis in adipose tissue, thus promoting lipogenesis [20, 47]. Therefore, the crux of the differences between the CIM and the EBM is two-fold. First, the CIM focuses on the endocrine response to the sources of dietary substrate, while the EBM focuses on the caloric content of the diet [20]. Second, the CIM focuses on fuel partitioning in the periphery (particularly adipose tissue), while the EBM focuses on the brain and its regulation of nutrient intake. However, the precise mechanisms still need to be resolved [19]. Both EBM and CIM stress the importance of diet; however, the EBM focuses on the quantity of calories, while CIM focuses on the quality of calories, specifically carbohydrates with a high glycemic index (GI) (i.e., higher insulin-stimulated response to carbohydrates). Carbohydrates produce higher insulin secretion levels, down-regulating the insulin receptor and leading to insulin resistance and altered signaling in the brain [48, 49]. On the other hand, restriction of such carbohydrates would result in lower insulin levels, reduced fat storage and increased lipolysis, and resultant weight loss. Indeed, increasing insulin promotes weight accrual in humans [50]; as demonstrated by type 1 diabetes (deficient insulin production); one of the cardinal symptoms of the disorder is weight (especially adipose tissue) loss, while insulin supplementation rapidly increases weight gain and adiposity. Pima Indians who have high rates of obesity also have significantly higher fasting insulin and display a higher amplitude insulin response to a glucose load [51].

UPF in the Western diet may also induce nutritional insufficiencies detrimental to the brain, resulting in a lack of critical nutrients vital for neurotransmitter function, cognition, mood, sleep and optimal neurodevelopment [52, 53]. Thus, the Western UPF-rich diet may play an essential role in the CIM model of obesity, as it does for the EBM model.

## The Energy Reduction-Oxidation Model (REDOX)

**Fig. 1:**
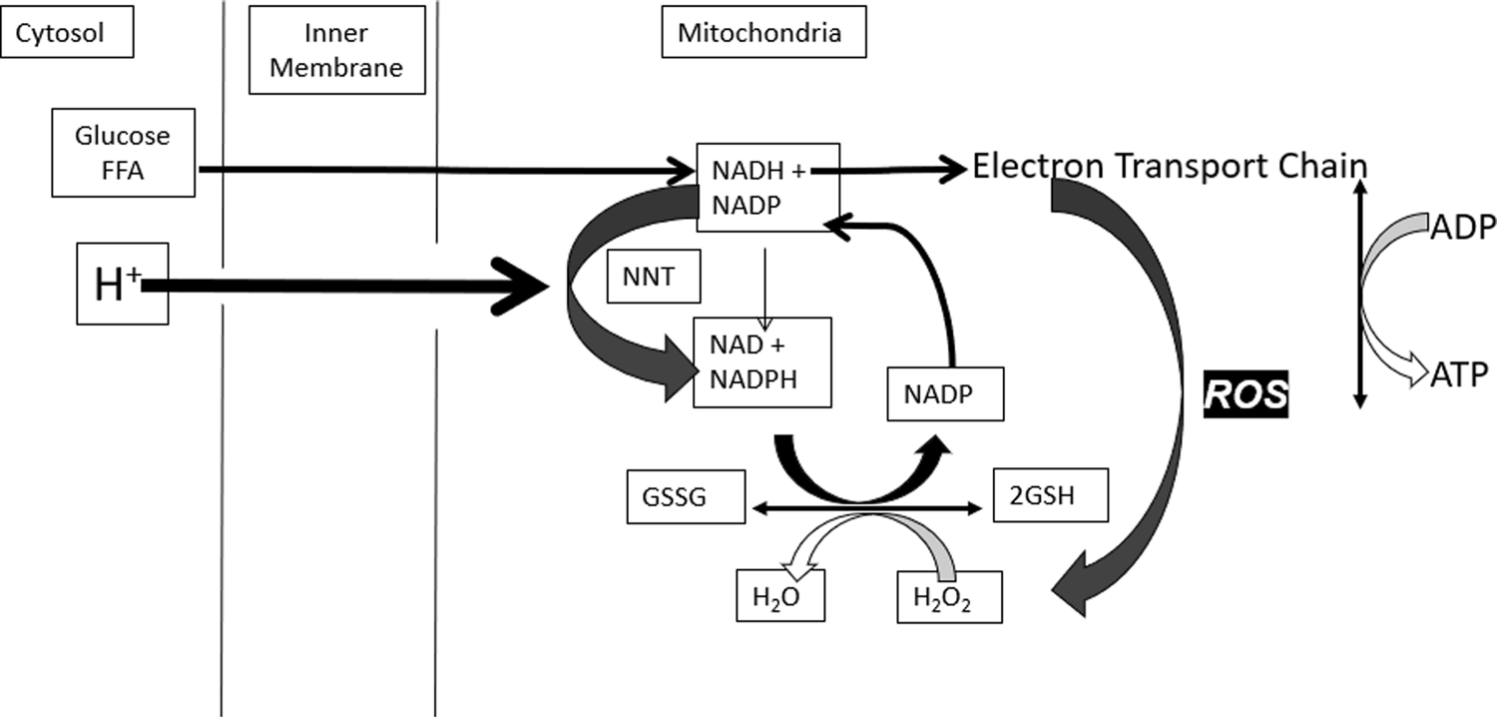
Mitochondrial NADH and NADPH: roles in energy and ROS metabolism. Fuel-generated NADH donates electrons to drive the ETC to convert ADP to ATP and generate ROS. This NADH is also converted to NADPH essential to remove ROS via NNT and peroxidases.

Mitochondria are the primary site of cellular ATP or energy production. Reactive oxygen species (ROS) is a natural signal that all mitochondria generate when energy needs have been met and fuel is still available. The value of this signal is to promote fuel storage via stimulation of insulin secretion. The short-lived signal stops when fuel has been stored and is no longer excessive. The mechanisms involved are biochemically complex: metabolism of glucose and fat generates reduced nicotinamide adenine dinucleotide (NADH) in the mitochondria. This fuel-derived NADH donates electrons to the electron transport chain (ETC) to maintain a high energy state or ATP/ADP ratio. When fuel supply exceeds the need for ATP production, elevated NADH produces ROS, an essential intracellular signal of fuel excess [21]. Excess fuel increases NADH and ROS rapidly in all cells, while lacking fuel decreases both. Rapid mitochondrial ROS removal requires NADPH, derived from glucose and fat metabolism. Redox reactants, therefore, comprise an energy-responsive communication system within each cell and cellular compartment [21, 54–56] (Fig. 1).

Although it is well-established that high levels of ROS can cause lipid peroxidation, protein denaturation, and cellular damage, the small increases in ROS that occur in response to acute fuel excess (assuming ATP adequacy) have significant positive cell-specific effects in metabolically active cells. ROS stimulates fat storage in adipocytes by regulating lipogenesis and lipolysis; the balance depends on the adipocyte’s hormonal milieu. For instance, elevated insulin stimulates LPL and inhibits lipolysis to drive TG synthesis, whereas catecholamines stimulate lipolysis. The hormonal milieu and ROS excess drive a net increase in TG stores in the presence of hyperinsulinemia or a net decrease when basal insulin is low [55]. In pancreatic ß-cells, ROS application externally or ROS generation internally stimulates insulin secretion [21, 57–59]. In the liver, a physiological increase in ROS reduces glucose output, whether of mitochondrial, cytosolic, or extracellular origin [55, 56]. ROS also acts in the hypothalamus to decrease food intake through effects on various neurons, including activation of pro-opiomelanocortin (POMC) neurons and suppression of agouti-related protein (AgRP)/neuropeptide Y (NPY) neurons [60]. Various hormones and nutrients also influence hypothalamic ROS generation [61]. Thus, the consequences of ROS production in response to excess glucose or fat supply are logical, synchronous, and coordinated: ß-cells release insulin to promote energy storage, adipocytes store triglycerides, hepatocytes stop gluconeogenesis, and neurons signal satiety.

Rapid ROS removal is achieved through catalase, glutathione, and thioredoxin systems. However, excess ROS production can exceed the capacity of thiol removal systems, resulting in oxidative damage to susceptible proteins and lipids, with resultant cell damage or death. The possibility that inadequate ROS removal systems could differentiate sensitive individuals from individuals that maintain average weight on similar diets has yet to be investigated.

**Fig. 2:**
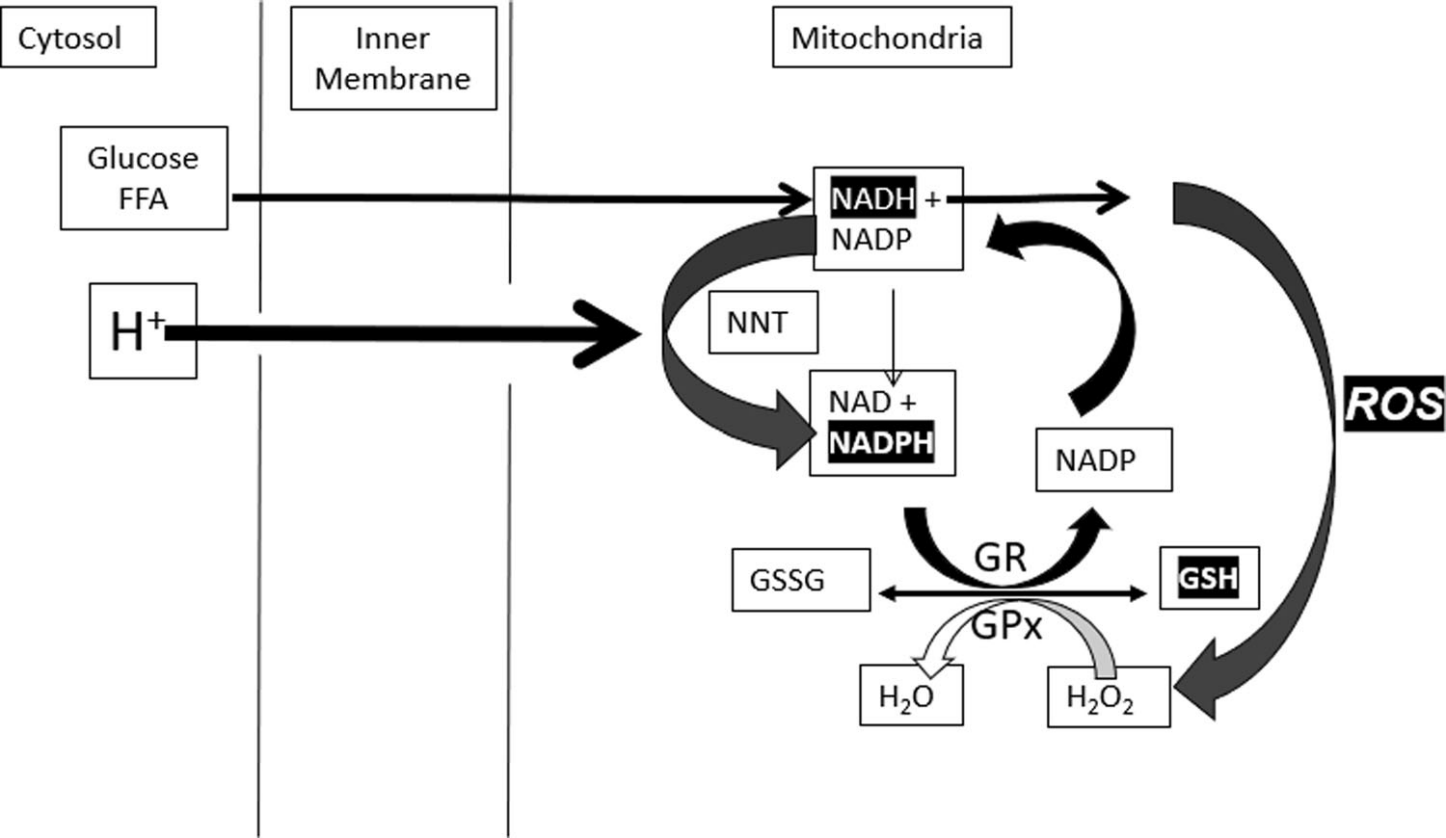
Coordinated oscillations GSH due to ROS production and removal. ROS induces oscillations in GSH that convert ROS to H_2_O (via GPX) NADPH to restore GSSG to GSH (via GR), NADH to restore NADPH (via NNT), membrane potential (via proton-driven NNT) and O_2_ (via ETC) to restore the proton gradient.

People with obesity [62] and those who consume large quantities of UPF [63] appear to have lower antioxidant capacity, including superoxide dismutase, glutathione peroxidase, and catalase, compared to people of normal weight [64–66]. If this results from insufficient antioxidant capacity, it would be expected to increase oxidative stress. Normal energy-dependent mitochondrial ROS signals are transient, and ROS removal requires NADPH produced from NADH via nicotinamide nucleotide transhydrogenase (NNT) (Fig. 2). Since flux through NNT is driven by the proton gradient, it decreases the mitochondrial membrane potential, ultimately stimulating restoration by the ETC [67–69]. This “proton leak” is readily determined by measuring oxygen consumption when ATP production is inhibited by oligomycin [68, 69].

Since excess nutrient consumption, the consumption of UPF and exposure to obesogens can all lead to an increase in ROS, the timing of the increase in ROS corresponds to the increase in the rate of obesity which correlates to excess nutrient consumption, UPF and obesogens.

Additional support for the REDOX model comes from data showing that enlarged adipocytes in obesity are associated with chronic low-grade inflammation in adipose tissue and increased oxidative stress [70]. Adipose tissue induces the synthesis of pro-inflammatory cytokines (TNF-α, interleukins IL-1 and IL-6), thereby promoting additional ROS generation by macrophages and monocytes [70]. Excessive fat accumulation in people with obesity leads to increased circulating free fatty acid levels, which promote higher fat oxidation and increased ROS [71].

It is also essential to be aware that ROS impacts thousands of proteins containing susceptible sulfhydryl groups on cysteine residues [72]. These proteins, in turn, regulate cellular signal transduction, including endocrine secretion and energy homeostasis [73].

Weight maintenance has been well-documented to involve variations in energy efficiency [39]. We suggest that the “leak” resulting from flux through the mitochondrial NNT is a positive and critical element in maintaining stable body fuel stores and regulating energy efficiency [67, 74]. Thus, excess fuel generates ROS as a signal. Subsequently, ROS removal stimulates flux through NNT and “wastes” energy when fuel is plentiful but not when resources are scarce or mitochondrial membrane potential is low. ROS removal also depends on thiol availability and rapid restoration of reduced thiol. We propose that overwhelming this ROS handling mechanism to compensate for caloric variation will diminish energy-wasting capability, sustain elevated ROS levels, and promote oxidative damage and metabolic dysregulation.

Another possible mechanism of REDOX metabolites influencing obesity is through epigenetic changes [75]. Alterations in the human epigenome are seen during nutritional privation or deprivation alterations, especially during the fetal or neonatal period [76]. Increased ROS formation has been demonstrated to increase methylation status in some tissues [77] and ROS may alter adipose tissue differentiation during development [78]. From a mechanistic standpoint, weight gain can be mitigated by increasing the amount of dietary folic acid, which reduces ROS formation [79] and gene methylation [80].

Excess fuel stimulates ROS, leading to insulin secretion (CIM model), promoting fat storage and altering appetite (EBM model). The link to obesogens is based on the observation that obesogens cause oxidative stress which is a consequence of excess ROS or the failure to remove ROS adequately. Thus, we hypothesize that excess fuel alone or combined with obesogens generates toxic amounts of ROS that cause damage—changes in either pyridine nucleotides or ROS impact redox. Our model hypothesizes that such linked changes in ROS and redox, provide a common mechanism by which each model leads to obesity.

## The Obesogen Model (OBS)

Obesogens are ingested or internalized chemicals that alter energy metabolism, increasing adiposity. Many act via alterations in endocrine signaling. They disrupt signaling pathways (e.g., hormone receptors, transcription factors, ROS) in various cell types and tissues that regulate energy intake and expenditure, nutrient handling, and adiposity. Indeed, they have been shown to act during development in animal models to disrupt adipose tissue development via increases in number, size, location, and function. They also alter the control of food intake and metabolic rate via effects on the pancreas, adipose tissue, liver, GI tract, brain and/or muscle, thereby altering the programming of the setpoint or sensitivity for developing obesity later in life [18].

As noted above, obesity can start *in utero* due to altered nutrition, the Developmental Origins of Health and Disease [81]. This same paradigm holds for obesogens: development is the most sensitive time for obesogen exposures to alter the epigenetic programming of developing metabolic tissues leading to tissues that “look” normal but have altered epigenetic profiles, leading to increased sensitivity to weight gain later in life [18, 82, 83]. Some characteristics of obesogen action during development include that subtle epigenetic changes may be detectable at birth but their effects may not be apparent until later in life e.g., a latency between exposure and weight gain which may last from months to decades, effects will likely be sex specific, the effects of developmental exposure to obesogens may not be apparent without a challenge or “second hit” later in life [18]. Thus, many studies of obesogen action focus on developmental exposure and effects on weight gain later in life. Obesogens can also act throughout the lifespan, where in most cases the effects may not be permanent, and across generations; transgenerational epigenetic inheritance [84, 85].

Obesogens can be natural (e.g., metals, viruses), anthropogenic prescription drugs, environmental (insecticides, plastics, household chemicals, particulate matter), or food components (fructose, *trans*-fats, preservatives, emulsifiers) [18, 86]. Obesogens include solvents (polychlorinated biphenyls (PCBs)); pesticides (e.g., dichlorodiphenyltrichloroethane (DDT), chlorpyrifos, diazinon, permethrin, neonicotinoids); non-stick coatings (e.g., per- and polyfluorinated substances (PFAS)); clothing and furniture protectants (e.g., polybrominated diphenyl ethers (PBDEs), organophosphate flame retardants (OPFRs)); food preservatives/additives/emulsifiers (e.g., parabens, monosodium glutamate, carboxymethylcellulose, 3-tert-butyl-4-hydroxyanisole (3-BHA)); personal care products (e.g., phthalates, parabens); plastics (e.g., phthalates, bisphenols); resins and can linings (e.g., bisphenols); and air pollutants (e.g., polycyclic aromatic hydrocarbons (PAHs), fine particulate matter (PM_2.5_)) [87]. Some pharmaceutical drugs [88, 89] and early-life antibiotics can also be obesogens. Exposures can occur via air, water, food, skin contact or dust inhalation [90, 91].

**Fig. 3:**
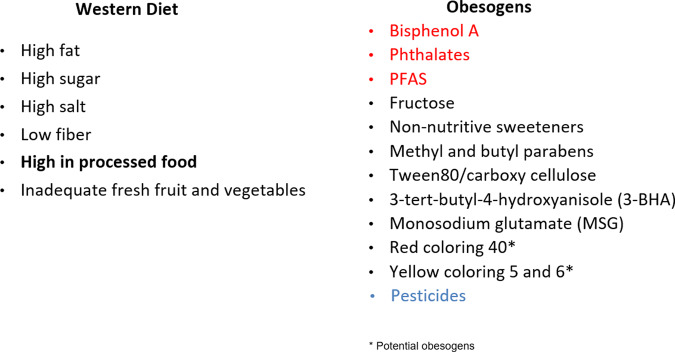
The Western ultra-processed food diet is obesogenic. The Western Diet (left panel) per se is obesogenic. In addition (right panel), chemicals in food packaging, such as can linings, can contain obesogens (red) which can leach into the food. Many food additives, preservatives, emulsifiers, and antioxidants are obesogens. Many fruits and vegetables are sprayed with pesticides, and some residues remain on them. Potential obesogens are those with only in vitro data. Reviewed in [87, 146, 147].

Obesogens include environmental chemicals that have arisen in the past 50–70 years with the first increase in the 1960s [92, 93], prior to the start of the increase in obesity in adults in the U.S. in the 1970s, and children a decade later as noted by NHANES studies [94, 95]. Everyone is now exposed to a variety of obesogenic chemicals. Human studies show that obesogens affect weight gain in various countries including Spain, Poland, Mexico, Denmark, Belgium, Greece among others indicating the global nature of the relation of obesogens to obesity [96–98]. Obesogens permeate our food supply (Fig. 3) and are often consumed unintentionally. They are also in our water supply and in the air we breathe.

Thousands of new chemicals have entered our food supply and environment since the obesity pandemic began. Hundreds of in vitro, animal, and human epidemiologic birth cohort studies show effects or associations between environmental chemicals and obesity, including systematic reviews and meta-analyses reviewed in [18, 96, 98–100]. While exposure is ubiquitous, the effects of obesogens vary depending on genetic susceptibility, age, sex, home and work location, personal habits, race, and diet. For example, African-Americans tend to be exposed to higher air pollutants, bisphenol A (BPA), phthalates, organochlorine pesticides, and PCBs because of their neighborhood environment, personal care products, and/or diet [101, 102].

Obesogens affect numerous metabolic endpoints across the lifespan, including adipocyte differentiation, adipocyte number, size, and function, lipid levels, the gut microbiome, food intake, energy expenditure, inflammation, and insulin resistance [18]. Similarly, obesogens can impact animals that share our environment [103–105]; perhaps obesogens can explain why even animals in captivity with controlled diets have gained weight over the last 25 years [43].

**Fig. 4:**
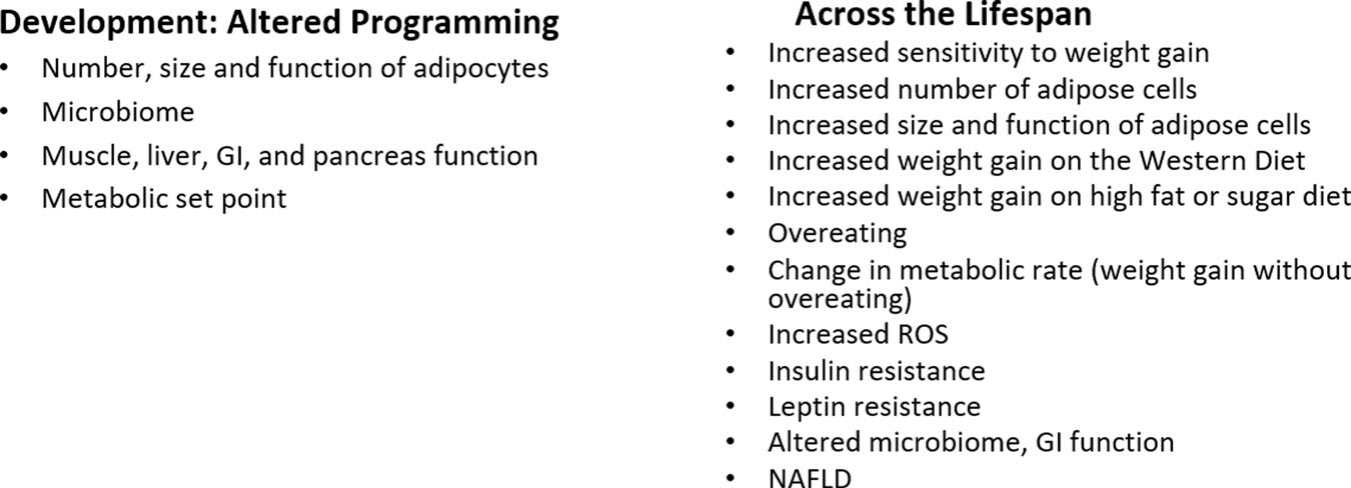
Integrating obesogen actions. Obesogens act during development to alter the programming of multiple tissues and processes that lead to increased sensitivity/susceptibility to weight gain across the lifespan [18, 82].

In September 2022, Healthy Environment and Endocrine Disruptor Strategies (HEEDS) held a workshop in Racine, WI to integrate the obesogen model into the thinking of mainstream basic, clinical obesity, and nutrition researchers [106]. A report from that meeting noted, “Based on the robust nature of the in vitro and animal model data on obesogens, the obesogen hypothesis/model of obesity should receive greater attention by the broader scientific community as a potential contributor to the obesity pandemic.” Fig. 4 overviews the OBS model. This workshop also outlined data gaps and needs for the OBS field. These include human data that show decreased obesogen exposure can improve metabolic health, leveraging clinical studies to establish causality, more experiments to understand the mechanism of obesogen action on the brain satiety and appetite centers and the hedonic, emotional eating center, and methods to determine the risk of obesity attributable to obesogens compared to diet, genetics and other factors.

## Proposed Integrated Model

### Integration of OBS and REDOX Models

In this perspective, we propose a testable composite model that first integrates the OBS and the REDOX model. We then propose an integration between this new OBS/REDOX model and the EBM and CIM models to create a unified model that can be used to assess the mechanisms responsible for obesity.

We posit that the obesity epidemic is due at least in part to exposure to exogenous obesogenic chemicals, some of which are in the UPF food supply, which in addition to affecting hormones, also induce an increase in ROS-mediated signals that alter metabolism.

This new OBS/REDOX model argues that obesogens that have entered our bodies recently, over the last 50–70 years, and cause obesity by interfering with endocrine receptor signaling (e.g., estrogen receptor, androgen receptor, glucocorticoid receptor, peroxisome proliferator-activated receptor (PPARγ), retinoid X receptor, thyroid hormone receptor (TR), chimeric antigen receptor, farnesoid X receptor, and aryl hydrocarbon receptor) [18], and hijack established redox signaling pathways to generate false and misleading information about energy status (e.g., ROS). Misinformation is driven by impacts on the oxidation-reduction (redox) potential of metabolites that circulate and communicate to organs throughout the body to modulate insulin secretion, fat storage and neural regulation of energy homeostasis [107–109].

**Fig. 5:**
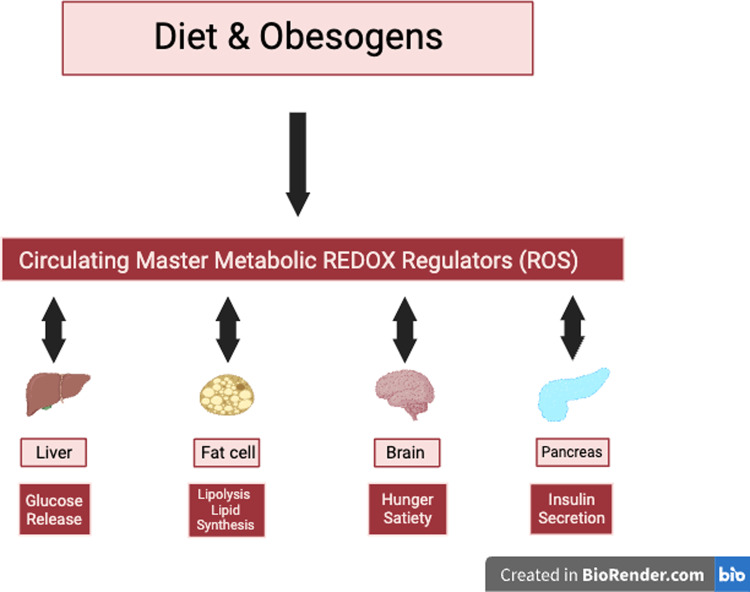
Integrating obesogen and ROS pathways. Obesogen exposure can interfere with the redox control of metabolism via stimulating or inhibiting enzymes that regulate ROS. The altered ROS signaling then affects a variety of obesity-related endpoints.

These obesogens can act during development via alterations of the concentration or timing of hormones and the concentration of ROS that controls epigenetic programming of development, resulting in tissues with altered gene expression. They can also work across the lifespan by changing hormone or ROS levels that trigger various components of metabolism [110]. The altered hormone signaling and increased ROS by obesogens results in all of the metabolic changes noted in obesity—increased insulin secretion, adipocyte differentiation, altered adipocyte size, number, inflammation and function, increased serum lipids, non-alcoholic fatty liver disease (NAFLD), altered microbiome, insulin and leptin resistance, increased food intake, altered resting metabolic rate, and reduced voluntary energy expenditure [18]. Variations in the capacity to scavenge ROS and exposure to obesogens may vary significantly and could explain variations in susceptibility to obesity. Figure 5 shows the interaction between diet, obesogens, ROS, and obesity.

The OBS-REDOX model presents a testable mechanistic concept. REDOX opens the door to the paradigm that new chemicals disseminated globally in the past half-century alter the cellular or circulating redox state and ROS pool resulting in inappropriate and false signals that subvert normal regulatory mechanisms (especially insulin) and possibly altering epigenetic programming of tissue gene expression in metabolic tissues.

The REDOX model predicts that ingesting ROS-generating obesogens will promote obesity by stimulating excess nutrient intake, insulin secretion, and fat storage or inhibiting energy efficiency adaptations. Such redox changes can be assessed by measuring the circulating thiol redox state (GSH, GSSG, cystine and cysteine) and increase in serum oxidized proteins [111, 112]. The OBS-REDOX model predicts that either a low-carb diet or the elimination of UPF will decrease ROS-mediated inappropriate signaling by reducing obesogen consumption and resultant ROS production.

What data support the concept that obesogens can alter ROS, thereby supporting this portion of the model? While ROS is not a typical endpoint assessed in obesogen experiments, virtually all obesogens, including tributyltin (TBT), BPA, arsenic, atrazine, cadmium, chlorpyrifos, di(2-ethylhexyl) phthalate (DEHP), perfluorooctanoic acid (PFOA), perfluorooctanesulfonic acid (PFOS), and 2,3,7,8-tetrachlorodibenzodioxin (TCDD) have been reported to target mitochondria, resulting in excessive ROS production, cell death, insulin resistance, and adipocyte differentiation and growth in a variety of in vitro and animal models, reviewed in [18]. Examples of the correlation between obesogens and ROS are numerous. PFAS are associated with ROS in animal and in vitro cell models [113]. TBT alters mitochondrial bioenergetics, which leads to excessive ROS production and insulin resistance [114]. BPA exposure to various cells and models induces oxidative stress and increases ROS [115]. Fine particulate matter air pollution (PM_2.5_) increases systemic oxidative stress, inflammation, and insulin resistance in mouse models of obesity [116, 117]. DEHP increases ROS and cell differentiation into adipocytes, decreasing adiponectin secretion in preadipocytes [118]. Some obesogens (e.g., DEHP and BPA) have been shown to promote oxidative stress and increase ROS levels in the HepG2 liver cell line [119], and PBDE-209 causes mitochondrial dysfunction in HepG2 cells [120].

Some obesogens (e.g., BPA and PCBs) also increase the levels of proinflammatory cytokines, such as TNF-α and IL-6, in cell cultures of adipocytes. For instance, exposure of 3T3-L1 preadipocytes to BPA promotes differentiation towards adipocytes and a proinflammatory state [121]. The obesogens TBT, DEHP, triclosan, and PM_2.5_ also cause inflammation in some animal models [18]. Therefore, obesogens may contribute to meta-inflammation development; a low-grade chronic inflammatory state observed in people with obesity is believed to constitute a link between obesity and related complications, including cardiovascular disease, type 2 diabetes mellitus, and dyslipidemia [5]. It should be noted that increases in ROS are often inferred but not directly measured; however, we measure increases in inflammation and oxidative stress, surrogate markers of ROS production [122].

The dichotomy of the EBM versus CIM has revolved around the Western diet’s quantity versus quality of macronutrients. The OBS-REDOX model notes that the Western diet is high in obesogens, which we propose as additional culprits. Because obesogens impact the redox state, consuming UPF (contaminated by obesogens) can increase inflammation and mitochondrial dysfunction, increase insulin secretion and insulin-mediated TG synthesis, and reduce insulin sensitivity [123] (Fig. 1).

The ability to detoxify excess ROS is likely to vary among tissues and individuals, although this process has yet to be investigated clinically. It is well-established that pancreatic ß-cells have a very low ability to scavenge ROS, making insulin secretion one of the earliest responses to excess ROS induced by either obesogens or excess nutrients. Hyperinsulinemia resulting from excess nutrients or dietary obesogens can increase adipose mass. Indeed, fat storage cannot occur without insulin, even in the ventromedial hypothalamus-lesioned animals [124]. Furthermore, ROS has been shown to stimulate insulin secretion directly in the absence of glucose [58, 125], and ROS removal reduces insulin secretion [59, 126].

### Application of the OBS-REDOX Model to other Models: overall integrated Model

The OBS/REDOX model is similar to the EBM in that it does not attribute causality to a particular nutrient or obesogen but can accommodate either when they induce a change in hormone function or redox potential. Changes in the redox state may occur via nutrients or obesogens in UPF foods, affecting hunger, satiety, insulin secretion, and/or adipose tissue storage.

As noted by Hall et al., further development of the EBM requires elucidation of the factors in the food environment that are most responsible for instigating obesity, the mechanisms by which these factors alter the brain circuits controlling food intake, and why some individuals are more susceptible to the development of obesity than others [19]. The OBS-REDOX model provides a focus on factors in the environment that could be responsible for instigating obesity (i.e., obesogens), the sites and mechanisms by which these chemicals might alter brain circuits controlling food intake (ROS and redox signals), and why some individuals are more susceptible to weight gain (differential exposures to obesogens, different ROS-scavenging capacities). Indeed, the obesogens TBT and BPA can alter satiety and appetite neurons; BPA, DEHP, and OPFRs can stimulate food intake; TBT and chlorpyrifos can stimulate weight gain with no change in food intake; TBT, BPA, butyl benzyl phthalate (BBzP), DDT permethrin, atrazine and chlorpyrifos show a more significant effect on weight gain on a HFD; BPA, DEHP, PBDEs, PAHs, triclosan and methylparaben alter the microbiome in animal models [18]; and PFAS results in a lower resting metabolic rate in humans [127, 128]—all endpoints that are implicit in the EBM. Furthermore, the tying in of the direct role of the diet-induced microbiome in insulin resistance and glucose intolerance could be due to ROS generation in the intestine, as diet-induced short-chain fatty acids are known inhibitors of ROS generation and action. It may play a specific role in preventing intestinal inflammation [129], thereby reducing hepatic insulin resistance, resulting in improvements in glucose tolerance, diabetes, and metabolic syndrome unrelated to changes in weight [129].

Secular trend data show that the current increase in obesity has occurred without an increase in food intake [3]. Transgenerational epigenetic inheritance of obesity [99, 130] has been demonstrated in rodent models for the obesogens TBT, BPA, phthalate-BPA mixture, and DDT. If transgenerational epigenetic inheritance for obesity were documented in humans, it could easily be responsible for weight gain without increased food intake in the current generation. Obesity in the parental generation could alter programming in the offspring, resulting in altered metabolic regulation. Weight gain could occur without concomitant overeating, which could account for the current increase in obesity without an increase in food intake, thus solving one of the perplexing conundrums of the EBM.

Like the EBM, the CIM does not address the mechanism for altering appetite control of the body weight set point. Indeed, it does not even agree that a set point exists. However, hyperinsulinemia resulting from excess dietary nutrient consumption can stimulate increased adipose mass, and the resulting insulin can antagonize leptin signal transduction at the POMC neuron, driving increased energy intake and decreased energy expenditure [131].

Since increased insulin is the central tenet of the CIM, how do obesogens stimulate insulin secretion? ROS has been shown to directly stimulate insulin secretion without a glucose stimulus [58], whereas ROS removal prevents insulin secretion [57]. In animal studies, many obesogens stimulate ROS and insulin release, including BPA, bisphenol S (BPS), bisphenol F (BPF), DEHP, cadmium, nonylphenol, triphenyl tin, TBT, PCBs, dioxin, mercury, and arsenic, reviewed in [132–135]. Additionally, BPA, DEHP, PM_2.5_, PFOS, atrazine, cadmium, permethrin, imidacloprid insecticides, OPFRs, chlorpyrifos, tolyfluanid, dibutyltin, fructose and the Western diet all lead to insulin resistance and hyperinsulinemia, which stimulate weight gain [18, 136–144].

The combined OBS/REDOX model predicts that a low-carb diet will decrease ROS-mediated inappropriate signaling by lowering glucose-induced insulin secretion and reducing consumption of food-borne obesogens. Thus, the OBS/REDOX model can explain the beneficial clinical effects of diets that decrease UPF and/or carbohydrate consumption, thus supporting the CIM [145].

**Fig. 6:**
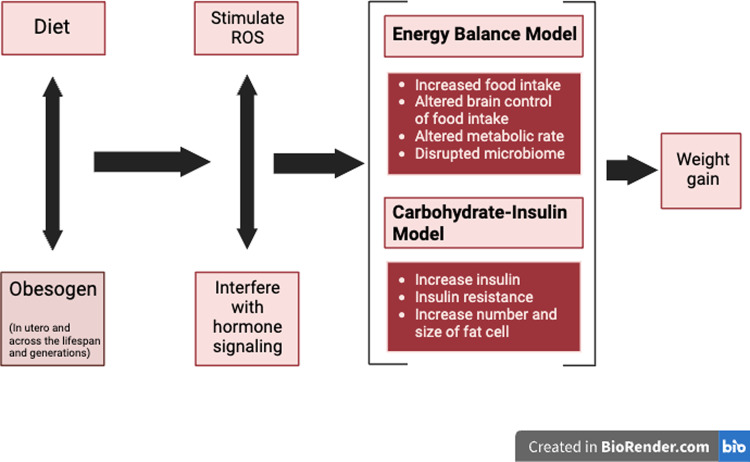
The OBS/REDOX model contributes to a unifying theory for the global rise in obesity. Obesogen exposure *in utero* and across the lifespan results in false hormonal and ROS signaling leading to altered metabolism. The endpoints affected by OBS/REDOX are the endpoints that are proposed to be altered by the EBM and CIM models. Thus, the OBS/REDOX model integrates all the models and serves as a unifying concept for the mechanisms responsible for the obesity pandemic.

The OBS/REDOX model accommodates aspects of both the EBM and CIM models, integrating them into one model where obesogen exposure can lead to ROS, defective energy utilization, and increased food intake. The proposed integrated model is shown in Fig. 6 and overviewed in Box 1.

Differentiating the relative contributions of obesogens, UPF, and carbohydrate-mediated hyperinsulinemia will require a comparison of the usual dietary constituents with identical but obesogen-free macronutrients, identification of obesogens present in humans at concentrations that generate ROS or redox changes, and determination of the effect of their removal on the circulating redox state in human subjects. Such studies have yet to be performed.

Box 1 Overview of an integrated model of obesity We propose two components to the obesity model: diet (especially the Western diet) and obesogens. Diet stimulates overeating, leading to weight gain. It can also stimulate insulin secretion, resulting in the disposition of fat. Obesogen exposure during development, *in utero* and early life alters the programming of metabolism, resulting in an altered set point for gaining weight: weight is gained more easily due to altered gene expression in the brain, adipose tissue, pancreas, GI tract and liver. Obesogens also stimulate misleading or false signals throughout life via alterations in hormone signaling pathways and increasing ROS. The altered hormone signaling pathways can lead to increased fat tissue, abnormal fat cell size, number and function, altered fat cell growth, increased insulin secretion, altered hypothalamic satiety and appetite neurons (the homeostatic pathway), NAFLD, leptin and insulin resistance, altered microbiome and inflammation: effects seen in and that support both EBM and CIM.

## Summary

Obesity is a multifactorial disease. Despite decades of research, searching for a single etiologic agent, target, hypothesis, pathophysiology, or magic bullet model has yet to be successful. While altered nutrition during pregnancy is very clearly a primary risk factor for postnatal weight gain due to developmental programming, neither the CIM nor EBM models focus on this aspect of the lifespan. On the other hand, the REDOX-OBS model focuses on the effects of obesogenic chemical exposures during development and how they send false autocrine and endocrine signals in metabolic tissues, increasing the sensitivity or susceptibility to weight gain later in life and even across generations. We have proposed a testable OBS-REDOX model consistent with aspects of the two major obesity models: EBM and CIM. Misleading cellular signals will stimulate food consumption, insulin release, and fat storage even when fuel is not excessive. The OBS-REDOX model provides data supporting changes in food intake and alterations in energy efficiency and storage.

A recent review noted that the debate between competing models could be more productive, and the field should focus more on establishing mechanistic insights leading to actionable interventions [47]. We agree; thus, we propose that all the current models make essential contributions to understanding the pathogenesis of the obesity pandemic. We provide an integrated model that can explain developmental programming and effects across the lifespan and generations, an altered metabolic set point, alterations in mitochondrial efficiency, and signals across metabolic tissues that convey a modified nutritional state. Our proposal (Box 1) is not that obesogen exposures per se are the sole cause of the obesity pandemic but that via effects on gene expression and ROS, obesogens alter the function of metabolic tissues such that people are more sensitive to diet-induced weight gain and less sensitive to weight loss.

The acceptance of this integrated model will focus on preventing obesity by reducing exposures to obesogens *in utero* and early life and throughout the lifespan. These include eating fresh organic foods, avoiding UPF, avoiding plastics for storing or heating food, using fragrance-free products, avoiding nonstick cookware, and using purified drinking water (for details, see www.ewg.org). Ultimately, regulatory and policy action will be needed to reduce the production of obesogenic chemicals.

## Future directions

We hope this model will result in an improved understanding of the etiologies of obesity, leading to improved intervention and prevention strategies. High-throughput screening systems for obesogen-induced effects on metabolic function are needed to allow definitive identification of harmful chemicals and their effective concentration range that would necessitate their removal from the environment. Understanding specific mechanisms, proteins, and pathways connecting obesogen exposures to the REDOX model are also needed. Documentation that EBM, CIM, or obesogens increase the thiol oxidation state measurable in the blood is essential to validating our hypothesis. The second step would involve determining the reversibility of weight gain by scavenging ROS, restoring the normal oxidation state, and/or reducing exposure to obesogens. The development of biomarkers of obesogen exposure, perhaps epigenetic or gene expression changes in metabolic tissues, action as well as ROS concentrations and mechanisms, including changes in extracellular ratios of pyruvate, acetate, and thiols, and their effect on intracellular redox and function, will aid in these endeavors. Finally, we have retrospective data to implicate obesogens and ROS in the pathogenesis of obesity. Still, we must prove causation. Therefore, future clinical trials should be designed to validate prospectively that obesogen exposure leads to alterations in ROS/oxidative stress biomarkers which can lead to weight and metabolic parameter changes [127, 128].
